# Discovery of *N*-(1-(3-fluorobenzoyl)-1*H*-indol-5-yl)pyrazine-2-carboxamide: a novel, selective, and competitive indole-based lead inhibitor for human monoamine oxidase B

**DOI:** 10.1080/14756366.2020.1800666

**Published:** 2020-08-04

**Authors:** Ahmed Elkamhawy, Sora Paik, Hyeon Jeong Kim, Jong-Hyun Park, Ashwini M. Londhe, Kyeong Lee, Ae Nim Pae, Ki Duk Park, Eun Joo Roh

**Affiliations:** a College of Pharmacy, Dongguk University-Seoul, Goyang, Republic of Korea; b Department of Pharmaceutical Organic Chemistry, Faculty of Pharmacy, Mansoura University, Mansoura, Egypt; c Chemical Kinomics Research Center, Korea Institute of Science and Technology (KIST), Seoul, Republic of Korea; d Convergence Research Center for Diagnosis, Treatment and Care System of Dementia, Korea Institute of Science and Technology (KIST), Seoul, Republic of Korea; e Department of Biotechnology, Yonsei University, Seoul, Republic of Korea; f Division of Bio-Medical Science & Technology, KIST School, Korea University of Science and Technology, Seoul, Republic of Korea; g KHU-KIST Department of Converging Science and Technology, Kyung Hee University, Seoul, Republic of Korea

**Keywords:** Monoamine oxidase B, carboxamide, MAO-B inhibitor, microwave synthesis, molecular modelling

## Abstract

Herein, two new series of *N*-substituted indole-based analogues were rationally designed, synthesized *via* microwave heating technology, and evaluated as noteworthy MAO-B potential inhibitors. Compared to the reported indazole-based hits **VI** and **VII**, compounds **4b** and **4e** exhibited higher inhibitory activities over MAO-B with IC_50_ values of 1.65 and 0.78 µM, respectively. When compared to the modest selectivity index of rasagiline (**II**, a well-known MAO-B inhibitor, SI > 50), both **4b** and **4e** also showed better selectivity indices (SI > 60 and 120, respectively). A further kinetic evaluation of the most potent derivative (**4e**) displayed a competitive mode of inhibition (inhibition constant (*K*_i_)/MAO-B = 94.52 nM). Reasonable explanations of the elicited biological activities were presented *via* SAR study and molecular docking simulation. Accordingly, the remarkable MAO-B inhibitory activity of **4e** (*N*-(1-(3-fluorobenzoyl)-1*H*-indol-5-yl)pyrazine-2-carboxamide), with its selectivity and competitive inhibition, advocates its potential role as a promising lead worthy of further optimization.

## 1. Introduction

Parkinson’s disease (PD), a neurodegenerative disorder with no currently available cure, is characterised by the impairment of motor function caused by the death of dopaminergic neurons in the substantia nigra pars compacta. Thus, the available therapies for ameliorating Parkinson’s symptoms mostly target dopamine depletion in the brain^1–6^. Monoamine oxidase B (MAO-B), which is localized on the outer membrane of mitochondria (mainly in the liver and brain), catalyzes the oxidative deamination of monoamine neurotransmitters such as dopamine which results in hydrogen peroxide (H_2_O_2_) production leading to oxidative stress and neuronal cell death^7–9^. Recently, MAO-B has been reported to be highly expressed in the substantia nigra of PD patients. Thus, inhibition of MAO-B is effective in alleviating the symptoms of PD patients. Indeed, several substances that can effectively ameliorate emerging motor symptoms of PD are being used nowadays by physicians, amongst them levodopa, dopamine agonists, and MAO-B inhibitors. However, mainly due to their favourable safety profile in addition to their putative neuroprotective capabilities, MAO-B inhibitors may constitute a preferable therapeutic option for early PD^10^^,^^11^.

**Figure 1.**
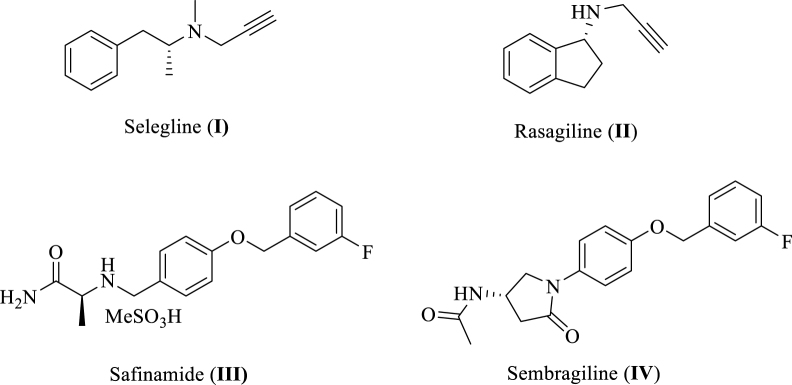
Chemical structures of well-known MAO-B inhibitors.

MAO-B is inhibited by different potent agents such as selegiline (**I**, Figure 1), rasagiline (**II**), safinamide (**III**), and sembragiline (**IV**), the most commonly used therapies for PD and Alzheimer’s disease (AD) *via* blocking dopamine degradation in the nigrostriatal pathway. However, numerous undesirable side effects of these inhibitors have been confirmed in long-term treatment of PD such as hallucinations, headaches, production of neurotoxic metabolites, sodium channel blockade, calcium channel modulation, and inhibition of stimulated release of glutamate^12–24^. Not only for that reason, but also since there is no disease-modifying treatment has been discovered yet for PD, there is still a vital need to develop novel selective MAO-B inhibitors as promising therapeutically active candidates for PD patients. Hence, this encouraged our institute to launch a discovery project aiming at identification of novel promising MAO-B inhibitors. As a result, various active small molecules have been recently reported including unsaturated ketones^25^, oxazolopyridines and thiazolopyridines^26^, biaryl derivatives^27^, in addition to indole-substituted benzothiazoles and benzoxazoles^28^. Moreover, other research groups reported diverse MAO-B inhibitors belonging to different chemical scaffolds such as chalcones, pyrazoles, chromones, coumarins, xanthines, isatin derivatives, thiazolidindiones, (thiazol-2-yl)hydrazones, 1,5-diphenylpenta-2,4-dien-1-ones, in addition to indazole- and indole-5-carboxamides^29–34^.

**Figure 2.**
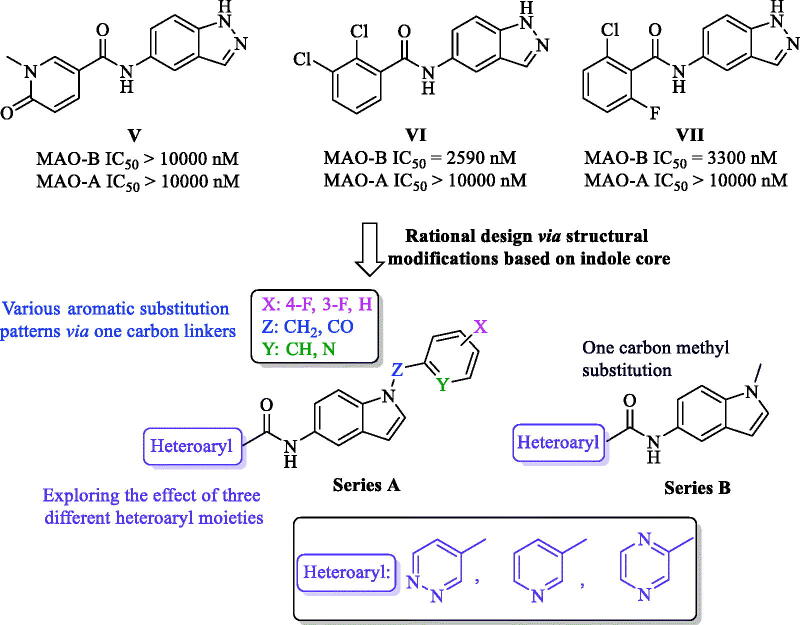
Rational design of the newly synthesized analogues (series A and B).

In the past few years, the indole moiety attracted the attention of medicinal chemists as a biologically accepted pharmacophore possessing wide spectrum of biological activities^35^^,^^36^. However, only few indole-based MAO-B inhibitors with high efficacy and promising safety profile were found in literature such as indole-based chalcones, indole-5-carboxamides, in addition to indole-substituted benzothiazoles and benzoxazoles^28^^,^^33^^,^^37^^,^^38^. One of these studies was carried out by Tzvetkov *et al* who preliminary prepared some indazole-based compounds (**V**, **VI**, and **VII**, Figure 2) to be evaluated over human MAO-B. While compound **V** was totally inactive, the biological results of **VI** and **VII** (MAO-B IC_50_=2590 and 3300 nM, respectively), coupled with their moderate selectivity values, were promising enough to pursue further structural modifications based on incorporation of a reverse amide linker to dramatically increase the activity and afford new highly potent indazole- and indole-5-carboxamides^33^. However, in their report, they mainly focussed on development of free NH indazole-based analogues with only two potent derivatives belonging to the indole scaffold. Accordingly, the current study took the optimization task for compounds **VI** and **VII** as a starting point towards novel N-substituted indole-based derivatives with potential inhibition against MAO-B.

The docking model of the most potent indazole derivative discovered by Tzvetkov *et al* proposed the binding site of MAO-B having some space remains unoccupied and, in addition, the indazole free NH was predicted not to be incorporated in any type of H-bond interaction with the binding site. By searching literature, the scaffold modification of compounds **VI** and **VII** into indole core retaining the same original amide linker at position 5 was found to be a completely new approach and not to be yet studied. Inspired by these observations, diverse chemical structural alterations on compounds **VI** and **VII** were planned as depicted in Figure 2; two novel series were designed *via* replacing the indazole core with indole scaffold while keeping the original amide spacer of **VI** and **VII** into 5-position of the indole, which have, to the best of our knowledge, not been reported so far. In addition, aiming to design our derivatives armed with a potential capability to bind into the predicted unoccupied space (in the binding site of MAO-B reported by Tzvetkov *et al*), the free NH of the indole moiety was substituted by one carbon spacer (Z = CH_2_ or CO) connecting the indole scaffold to various aromatic substitution patterns in series A, while in series B, the indole nitrogen was linked to one carbon methyl group. Moreover, we incorporated three different six-membered aromatic heterocycles (pyridine, pyridazine, or pyrazine), not only anticipated to interact with the binding site of MAO-B in a similar fashion to the substituted aryl moieties in **V** and **VI**, but also, targeting possible extra-interaction(s) owing to the heteroatom(s) in these six-membered systems. Accordingly, in this study, we present the complete synthetic routes of the newly synthesized compounds through microwave heating technology, the enzymatic inhibitory activities and selectivity values over MAO enzymes, structure–activity relationship (SAR), a detailed kinetic study, in addition to computational docking models of the most active ligands with MAO enzymes.

## 2. Materials and methods

### 2.1. Chemistry

*General:* All solvents and reagents were obtained from commercial suppliers and used without further purification. Microwave-assisted synthetic procedure was applied in a Biotage Initiator + apparatus operating in single mode; the microwave cavity producing controlled irradiation at 2.45 GHz (Biotage AB, Uppsala, Sweden). The reactions were carried out in sealed vessels by employing magnetic stirring with maintaining the desired temperature for the programmed time period. TLC was performed using glass sheets pre-coated with silica gel 60 F_254_ purchased by Merck (Kenilworth, NJ). The NMR spectra were obtained on Bruker Avance 400 (Billerica, MA). Column chromatography was carried out on Merck Silica Gel 60 (230–400 mesh) (Kenilworth, NJ). High-resolution spectra were performed on Waters ACQUITY UPLC BEH C18 1.7 μ–Q-TOF SYNAPT G2-Si High Definition Mass Spectrometry.

#### 2.1.1. General procedure for synthesis of compounds 2a–c

For compounds **2a**–**2c**; NaH (60% in oil, 48.0 mg, 1.2 mmol) was added to a solution of 5-nitroindole (**1**, 162.2 mg, 1.0 mmol) dissolved in DMF (11 mL) at 0 °C. The reaction mixture was stirred at 0 °C for 10 min. The appropriate reagent (1.0 mmol; 4-fluorobenzyl bromide for **2a**, 3-fluorobenzoyl chloride for **2b**, and 2-(chloromethyl)pyridine hydrochloride for **2c**) was added to the mixture. The mixture was then stirred at 100 °C for 24 h, cooled to room temperature, and partitioned between EA and water using aqueous NaHCO_3_. The organic layer was dried over anhydrous Na_2_SO_4_ and concentrated under reduced pressure. Finally, the residue was purified by column chromatography (SiO_2_, EA/*n*-Hex) to get the target intermediate.

##### 2.1.1.1. 1-(4-Fluorobenzyl)-5-nitro-1H-indole (2a)

Yellow solid, yield: 91%, mp: 126.4–127.0 °C, ^1^H NMR (400 MHz, CDCl_3_) *δ* 5.34 (s, 2H), 6.74 (s, 1H), 7.03 (d, *J* = 8.1 Hz, 2H), 7.08 (s, 2H), 7.28 (d, *J* = 10.6 Hz, 2H), 8.08 (d, *J* = 8.6 Hz, 1H), 8.60 (s, 1H). Reported^39^.

##### 2.1.1.2. (3-Fluorophenyl)(5-nitro-1H-indol-1-yl)methanone (2b)

Yellowish white solid, yield: 67%, mp: 151.0–152.0 °C, ^1^H NMR (400 MHz, CDCl_3_) *δ* 6.79 (d, *J* = 3.7 Hz, 1H), 7.35–7.40 (m, 1H), 7.47–7.50 (m, 2H), 7.54–7.60 (m, 2H), 8.25 (dd, *J* = 2.2 Hz, 9.1 Hz, 1H), 8.47–8.51 (m, 2H). ^13^C NMR (100 MHz, CDCl_3_) *δ* 109.24, 116.52, 116.53 (*J*_C–F_ = 24.1 Hz), 117.20, 119.86 (*J*_C–F_ = 21.1 Hz), 120.27, 125.06 (*J*_C–F_ = 3.0 Hz), 130.06, 130.69 (*J*_C–F_ = 5.0 Hz), 130.79, 135.29 (*J*_C–F_ = 8.1 Hz), 138.97, 144.55, 162.51 (*J*_C–F_ = 249.5 Hz), 167.08.

##### 2.1.1.3. 5-Nitro-1-(pyridin-2-ylmethyl)-1H-indole (2c)

Yellow solid, yield: 24%, ^1^H NMR (400 MHz, CDCl_3_) *δ* 5.49 (s, 2H), 6.76–6.80 (m, 2H), 7.20–7.23 (m, 1H), 7.33 (d, *J* = 9.0 Hz, 1H), 7.37 (d, *J* = 3.2 Hz, 1H), 7.59 (td, *J* = 1.6 Hz, 7.7 Hz, 1H), 8.08 (dd, *J* = 2.1 Hz, 9.1 Hz, 1H), 8.61 (d, *J* = 1.9 Hz, 2H). Reported^40^^,^^41^.

#### 2.1.2. Synthesis of 1-methyl-5-nitro-1H-indole (2d)

K_2_CO_3_ (55.3 mg, 0.24 mmol) was added to a solution of 5-nitroindole (**1**, 162.2 mg, 1.0 mmol) in DMF (5 mL). Dimethyl carbonate (DMC) (0.17 mL, 2.0 mmol) was added and the reaction mixture was refluxed for 3 h, then cooled to 4–5 °C, followed by adding ice-cold water to the mixture. Precipitated solid was filtered and washed with water. Yellow solid, yield: 83%, mp: 164.7–165.9 °C, ^1^H NMR (400 MHz, CDCl_3_) *δ* 3.85 (s, 3H), 6.66 (d, *J* = 2.8 Hz, 1H), 7.21 (d, *J* = 2.9 Hz, 1H), 7.33 (d, *J* = 9.0 Hz, 1H), 8.11 (d, *J* = 9.0 Hz, 1H), 8.57 (s, 1H). Reported^42^.

#### 2.1.3. General procedure of compound 3a–d

Each of compounds **2a**−**d** (1.0 mmol) was dissolved in EtOH/H_2_O (5/2), followed by adding NH_4_Cl (267.5 mg, 5.0 mmol) and iron powder (558.5 mg, 10.0 mmol). The reaction mixture was refluxed for 1 h and filtered on celite then washed with EtOH. The residue was extracted using EA, water and aqueous NaHCO_3_. The organic layer was dried over anhydrous Na_2_SO_4_ and concentrated under reduced pressure. The residue was purified by column chromatography (SiO_2_, EA/*n*-Hex) to get the target intermediate.

##### 2.1.3.1. 1-(4-Fluorobenzyl)-1H-indol-5-amine (3a)

Brown solid, yield: 80%, mp: 76.2–77.4 °C, ^1^H NMR (400 MHz, DMSO-d_6_) *δ* 4.49 (s, 2H), 5.27 (s, 2H), 6.18 (d, *J* = 2.8 Hz, 1H), 6.49 (dd, *J* = 1.8 Hz, 8.6 Hz, 1H), 6.69 (d, *J* = 1.6 Hz, 1H), 7.12 (t, *J* = 9.0 Hz, 3H), 7.20 (t, *J* = 8.5 Hz, 2H), 7.28 (d, *J* = 3.0 Hz, 1H). ^13^C NMR (100 MHz, DMSO-d_6_) *δ* 48.80, 99.98, 104.01, 110.64, 112.32, 115.65 (*J*_C–F_ = 21.1 Hz), 128.94, 129.41 (*J*_C–F_ = 8.0 Hz), 129.80, 130.07, 135.35 (*J*_C–F_ = 3.0 Hz), 142.00, 161.79 (*J*_C–F_ = 243.5 Hz). Reported^43^^,^^44^.

##### 2.1.3.2. (5-Amino-1H-indol-1-yl)(3-fluorophenyl)methanone (3b)

Orange solid, yield: 91%, mp: 91.7–92.9 °C, ^1^H NMR (400 MHz, DMSO-d_6_) *δ* 5.06 (s, 2H), 6.54 (d, *J* = 3.7 Hz, 1H), 6.68 (dd, *J* = 2.1 Hz, 8.7 Hz, 1H), 6.77 (d, *J* = 2.0 Hz, 1H), 7.19 (d, *J* = 3.7 Hz, 1H), 7.49–7.66 (m, 4H), 8.01 (d, *J* = 8.7 Hz, 1H). ^13^C NMR (100 MHz, DMSO-d_6_) *δ* 104.60, 109.35, 113.38, 116.07 (*J*_C–F_ = 23.1 Hz), 116.81, 118.88 (*J*_C–F_ = 21.1 Hz), 125.23 (*J*_C–F_ = 3.0 Hz), 128.06, 128.23, 131.36 (*J*_C–F_ = 8.1 Hz), 132.34, 137.14 (*J*_C–F_ = 7.0 Hz), 146.25, 162.17 (*J*_C–F_ = 245.5 Hz).

##### 2.1.3.3. 1-(Pyridin-2-ylmethyl)-1H-indol-5-amine (3c)

Light orange solid, yield: 72%, mp: 94.1–95.3 °C, ^1^H NMR (400 MHz, DMSO-d_6_) *δ* 4.51 (s, 2H), 5.38 (s, 2H), 6.22 (s, 1H), 6.49 (d, *J* = 8.2 Hz, 1H), 6.71 (s, 1H), 6.86 (d, *J* = 7.7 Hz, 1H), 7.07 (d, *J* = 8.5 Hz, 1H), 7.26–7.30 (m, 2H), 7.69 (t, *J* = 6.8 Hz, 1H), 8.54 (d, *J* = 3.5 Hz, 1H). ^13^C NMR (100 MHz, DMSO-d_6_) *δ* 51.69, 100.09, 104.01, 110.57, 112.34, 121.39, 122.91, 129.30, 129.76, 130.26, 137.45, 142.05, 149.55, 158.41. Reported^45^.

##### 2.1.3.4. 1-Methyl-1H-indol-5-amine (3d)

Brown solid, yield: 78%, mp: 103.0–103.9 °C, ^1^H NMR (400 MHz, DMSO-d_6_) *δ* 3.68 (s, 3H), 4.48 (s, 2H), 6.12 (d, *J* = 2.7 Hz, 1H), 6.56 (dd, *J* = 2.0 Hz, 8.6 Hz, 1H), 6.70 (d, *J* = 1.8 Hz, 1H), 7.09–7.12 (m, 2H). Reported^46^^,^^47^.

#### 2.1.4. General procedure of compounds 4a–l

To an MW vial, were successively added compound **3** (0.1 mmol), HATU (41.8 mg, 0.11 mmol), DIPEA (0.05 mL, 0.27 mmol), the appropriate heteroaryl carboxylic acid derivative (0.11 mmol) and DMF (12 mL) at room temperature. The MW vial was sealed and heated under MW conditions for 45 min at 116 °C. The reaction mixture was extracted using EA, water and brine. The organic layer was dried over anhydrous Na_2_SO_4_ and concentrated under reduced pressure. The residue was purified by column chromatography (SiO_2_, EA/*n*-Hex) to get the desired target compound.

##### 2.1.4.1. N-(1-(4-fluorobenzyl)-1H-indol-5-yl)pyridazine-4-carboxamide (4a)

Yellowish green solid, yield: 46%, mp: 212.6–213.1 °C, ^1^H NMR (400 MHz, DMSO-d_6_) *δ* 5.42 (s, 2H), 6.52 (d, *J* = 2.6 Hz, 1H), 7.15 (t, *J* = 8.8 Hz, 2H), 7.26 (t, *J* = 8.0 Hz, 2H), 7.41 (d, *J* = 8.0 Hz, 1H), 7.48 (d, *J* = 8.6 Hz, 1H), 7.54 (d, *J* = 2.8 Hz, 1H), 8.04 (s, 1H), 8.13 (d, *J* = 2.9 Hz, 1H), 9.48 (d, *J* = 4.7 Hz, 1H), 9.67 (s, 1H), 10.59 (s, 1H). ^13^C NMR (100 MHz, DMSO-d_6_) *δ* 48.89, 101.82, 110.57, 113.13, 115.80 (*J*_C–F_ = 21.1 Hz), 116.40, 124.92, 128.58, 129.53 (*J*_C–F_ = 8.1 Hz), 130.39, 130.99, 132.98, 133.59, 134.93 (*J*_C–F_ = 3.0 Hz), 149.47, 152.54, 161.89 (*J*_C–F_ = 243.5 Hz), 162.29.

##### 2.1.4.2. N-(1-(4-fluorobenzyl)-1H-indol-5-yl)pyrazine-2-carboxamide (4b)

Light yellow solid, yield: 73%, mp: 161.5–162.0 °C, HPLC purity: 6.02 min, 100%, ^1^H NMR (400 MHz, DMSO-d_6_) *δ* 5.42 (s, 2H), 6.51 (d, *J* = 3.0 Hz, 1H), 7.15 (t, *J* = 8.9 Hz, 2H), 7.26–7.30 (m, 2H), 7.46 (d, *J* = 8.8 Hz, 1H), 7.53–7.56 (m, 2H), 8.16 (d, *J* = 1.5 Hz, 1H), 8.82 (t, *J* = 1.5 Hz, 1H), 8.93 (d, *J* = 2.4 Hz, 1H), 9.32 (d, *J* = 1.2 Hz, 1H), 10.58 (s, 1H). ^13^C NMR (100 MHz, DMSO-d_6_) *δ* 48.88, 101.76, 110.48, 113.00, 115.78 (*J*_C–F_ = 21.1 Hz), 116.53, 128.55, 129.55 (*J*_C–F_ = 9.1 Hz), 130.27, 130.87, 133.48, 134.95 (*J*_C–F_ = 4.0 Hz), 143.65, 144.35, 145.86, 147.92, 161.60, 161.88 (*J*_C–F_ = 243.5 Hz). HRMS (ESI) *m/z* calcd. for C_20_H_15_FN_4_O [M + H]^+^: 347.1308. Found: 347.1306.

##### 2.1.4.3. N-(1-(4-fluorobenzyl)-1H-indol-5-yl)nicotinamide (4c)

White solid, yield: 53%, mp: 200.0–200.4 °C, ^1^H NMR (400 MHz, DMSO-d_6_) *δ* 5.42 (s, 2H), 6.50 (d, *J* = 3.0 Hz, 1H), 7.15 (t, *J* = 8.9 Hz, 2H), 7.25–7.28 (m, 2H), 7.44 (dd, *J* = 8.8 Hz, 17.8 Hz, 2H), 7.53 (d, *J* = 3.0 Hz, 1H), 7.56–7.59 (m, 1H), 8.04 (s, 1H), 8.31 (d, *J* = 8.0 Hz, 1H), 8.76 (d, *J* = 3.5 Hz, 1H), 9.13 (d, *J* = 1.4 Hz, 1H), 10.34 (s, 1H). ^13^C NMR (100 MHz, DMSO-d_6_) *δ* 48.87, 101.72, 110.42, 113.08, 115.77 (*J*_C–F_ = 22.1 Hz), 116.61, 123.93, 128.58, 129.51 (*J*_C–F_ = 9.1 Hz), 130.20, 131.37, 131.49, 133.39, 134.98 (*J*_C–F_ = 3.0 Hz), 135.78, 161.88 (*J*_C–F_ = 242.5 Hz), 164.06.

##### 2.1.4.4. N-(1-(3-fluorobenzoyl)-1H-indol-5-yl)pyridazine-4-carboxamide (4d)

Yellowish white solid, yield: 75%, mp: 196.7–196.9 °C, ^1^H NMR (400 MHz, DMSO-d_6_) *δ* 6.84 (d, *J* = 3.6 Hz, 1H), 7.44 (d, *J* = 3.6 Hz, 1H), 7.54–7.71 (m, 5H), 8.16–8.18 (m, 1H), 8.23 (s, 1H), 8.33 (d, *J* = 8.9 Hz, 1H), 9.52 (d, *J* = 4.9 Hz, 1H), 9.70 (s, 1H), 10.85 (s, 1H). ^13^C NMR (100 MHz, DMSO-d_6_) *δ* 109.54, 113.17, 116.26, 116.51, 118.57, 119.37 (*J*_C–F_ = 20.1 Hz), 125.01, 125.55 (*J*_C–F_ = 3.0 Hz), 129.45, 131.40, 131.45, 131.53, 132.75 (*J*_C–F_ = 5.0 Hz), 135.08, 136.57 (*J*_C–F_ = 7.04 Hz), 149.46, 152.58, 162.20 (*J*_C–F_ = 245.5 Hz), 162.75, 167.18.

##### 2.1.4.5. N-(1-(3-fluorobenzoyl)-1H-indol-5-yl)pyrazine-2-carboxamide (4e)

White solid, yield: 94%, mp: 221.3–222.2 °C, HPLC purity: 6.00 min, 100%, ^1^H NMR (400 MHz, DMSO-d_6_) *δ* 6.81 (d, *J* = 2.8 Hz, 1H), 7.43 (d, *J* = 3.0 Hz, 1H), 7.56 (t, *J* = 8.4 Hz, 1H), 7.61–7.67 (m, 3H), 7.86 (d, *J* = 8.9 Hz, 1H), 8.28 (d, *J* = 8.8 Hz, 1H), 8.32 (s, 1H), 8.85 (s, 1H), 8.96 (s, 1H), 9.34 (s, 1H), 10.85 (s, 1H). ^13^C NMR (100 MHz, DMSO-d_6_) *δ* 109.54, 113.16, 116.32, 116.37 (*J*_C–F_ = 23.1 Hz), 118.77, 119.36 (*J*_C–F_ = 20.1 Hz), 125.55, 129.33, 131.33, 131.49 (*J*_C–F_ = 8.1 Hz), 132.61, 134.97, 136.59 (*J*_C–F_ = 8.1 Hz), 143.72, 144.49, 145.65, 148.13, 162.11, 162.20 (*J*_C–F_ = 245.5 Hz), 167.13. HRMS (ESI) *m/z* calcd. for C_20_H_13_FN_4_O_2_ [M + H]^+^: 361.1101. Found: 361.1095.

##### 2.1.4.6. N-(1-(3-fluorobenzoyl)-1H-indol-5-yl)nicotinamide (4f)

White solid, yield: 57%, mp: 212.9–214.2 °C, ^1^H NMR (400 MHz, DMSO-d_6_) *δ* 6.82 (d, *J* = 3.6 Hz, 1H), 7.43 (d, *J* = 3.6 Hz, 1H), 7.56–7.73 (m, 6H), 8.23 (d, *J* = 1.5 Hz, 1H), 8.30 (d, *J* = 8.9 Hz, 1H), 8.35 (d, *J* = 7.9 Hz, 1H), 8.78–8.79 (m, 1H), 9.16 (d, *J* = 1.5 Hz, 1H), 10.58 (s, 1H). ^13^C NMR (100 MHz, DMSO-d_6_) *δ* 109.57, 113.07, 116.36 (*J*_C–F_ = 24.1 Hz), 116.38, 118.63, 119.34 (*J*_C–F_ = 21.1 Hz), 123.99, 125.51, 129.29, 131.12, 131.37, 131.49 (*J*_C–F_ = 8.1 Hz), 132.46, 135.64, 135.92, 136.63 (*J*_C–F_ = 7.0 Hz), 149.15, 152.55, 162.20 (*J*_C–F_ = 245.5 Hz), 164.47, 167.15.

##### 2.1.4.7. N-(1-(pyridin-2-ylmethyl)-1H-indol-5-yl)pyridazine-4-carboxamide (4g)

Yellow solid, yield: 85%, mp: 141.8–143.0 °C, ^1^H NMR (400 MHz, DMSO-d_6_) *δ* 5.52 (s, 2H), 6.54 (d, *J* = 2.7 Hz, 1H), 6.96 (d, *J* = 7.8 Hz, 1H), 7.28 (t, *J* = 5.3 Hz, 1H), 7.39–7.45 (m, 2H), 7.53 (d, *J* = 2.9 Hz, 1H), 7.71 (t, *J* = 6.9 Hz, 1H), 8.06 (s, 1H), 8.13–8.14 (m, 1H), 8.55 (d, *J* = 4.4 Hz, 1H), 9.49 (d, *J* = 5.1 Hz, 1H), 9.67 (s, 1H), 10.61 (s, 1H). ^13^C NMR (100 MHz, DMSO-d_6_) *δ* 51.70, 101.85, 110.57, 113.13, 116.44, 121.53, 123.10, 124.93, 128.55, 130.77, 131.01, 132.98, 133.84, 137.59, 149.48, 149.72, 152.55, 157.89, 162.29.

##### 2.1.4.8. N-(1-(pyridin-2-ylmethyl)-1H-indol-5-yl)pyrazine-2-carboxamide (4h)

Light yellow solid, yield: 39%, mp: 152.0–152.9 °C, ^1^H NMR (400 MHz, DMSO-d_6_) *δ* 5.51 (s, 2H), 6.52 (d, *J* = 3.0 Hz, 1H), 6.96 (d, *J* = 7.8 Hz, 1H), 7.26–7.29 (m, 1H), 7.41 (d, *J* = 8.8 Hz, 1H), 7.51–7.53 (m, 2H), 7.71 (td, *J* = 1.6 Hz, 7.6 Hz, 1H), 8.15 (d, *J* = 1.5 Hz, 1H), 8.54 (d, *J* = 4.6 Hz, 1H), 8.81 (d, *J* = 1.5 Hz, 1H), 8.92 (d, *J* = 2.4 Hz, 1H), 9.31 (s, 1H), 10.57 (s, 1H). ^13^C NMR (100 MHz, DMSO-d_6_) *δ* 51.70, 101.80, 110.47, 113.00, 116.56, 121.55, 123.09, 128.52, 130.67, 130.89, 133.74, 137.59, 143.66, 144.36, 145.87, 147.93, 149.71, 157.93, 161.62.

##### 2.1.4.9. N-(1-(pyridin-2-ylmethyl)-1H-indol-5-yl)nicotinamide (4i)

Light brown solid, yield: 38%, mp: 142.9–144.3 °C, ^1^H NMR (400 MHz, DMSO-d_6_) *δ* 5.52 (s, 2H), 6.52 (d, *J* = 2.9 Hz, 1H), 6.95 (d, *J* = 7.8 Hz, 1H), 7.29 (t, *J* = 4.9 Hz, 1H), 7.41 (s, 2H), 7.52 (d, *J* = 2.9 Hz, 1H), 7.56–7.59 (m, 1H), 7.70–7.74 (m, 1H), 8.05 (s, 1H), 8.31 (d, *J* = 7.8 Hz, 1H), 8.55 (d, *J* = 4.2 Hz, 1H), 8.76 (d, *J* = 3.5 Hz, 1H), 9.13 (s, 1H), 10.32 (s, 1H). ^13^C NMR (100 MHz, DMSO-d_6_) *δ* 51.71, 101.76, 110.41, 113.10, 116.65, 121.51, 123.08, 123.93, 128.55, 130.59, 131.37, 131.52, 133.64, 135.79, 137.58, 149.07, 149.70, 152.31, 157.96, 164.08.

##### 2.1.4.10. N-(1-methyl-1H-indol-5-yl)pyridazine-4-carboxamide (4j)

Yellowish green solid, yield: 28%, mp: 181.1–182.0 °C, ^1^H NMR (400 MHz, DMSO-d_6_) *δ* 3.81 (s, 3H), 6.45 (d, *J* = 3.0 Hz, 1H), 7.36 (d, *J* = 3.0 Hz, 1H), 7.47 (s, 2H), 8.04 (s, 1H), 8.15 (dd, *J* = 2.3 Hz, 5.3 Hz, 1H), 9.50 (dd, *J* = 1.0 Hz, 5.3 Hz, 1H), 9.69 (t, *J* = 1.0 Hz, 1H), 10.61 (s, 1H). ^13^C NMR (100 MHz, DMSO-d_6_) *δ* 33.03, 100.94, 110.08, 112.94, 116.15, 124.91, 128.20, 130.77, 130.94, 133.03, 134.35, 149.49, 152.53, 162.25.

##### 2.1.4.11. N-(1-methyl-1H-indol-5-yl)pyrazine-2-carboxamide (4k)

Brown solid, yield: 63%, mp: 172.5–174.0 °C, ^1^H NMR (400 MHz, DMSO-d_6_) *δ* 3.80 (s, 3H), 6.44 (s, 1H), 7.34 (s, 1H), 7.44 (d, *J* = 7.8 Hz, 1H), 7.61 (d, *J* = 7.1 Hz, 1H), 8.15 (s, 1H), 8.83 (s, 1H), 8.94 (s, 1H), 9.32 (s, 1H), 10.57 (s, 1H). ^13^C NMR (100 MHz, DMSO-d_6_) *δ* 33.01, 100.90, 110.00, 112.72, 116.22, 128.19, 130.67, 130.83, 134.26, 143.66, 144.34, 145.94, 147.90, 161.59.

##### 2.1.4.12. N-(1-methyl-1H-indol-5-yl)nicotinamide (4l)

White solid, yield: 22%, mp: 164.2–164.9 °C, ^1^H NMR (400 MHz, DMSO-d_6_) *δ* 3.80 (s, 3H), 6.43 (d, *J* = 2.9 Hz, 1H), 7.34 (d, *J* = 3.0 Hz, 1H), 7.42–7.49 (m, 2H), 7.56–7.60 (m, 1H), 8.03 (d, *J* = 1.2 Hz, 1H), 8.32–8.34 (m, 1H), 8.77 (dd, *J* = 1.4 Hz, 4.8 Hz, 1H), 9.15 (d, *J* = 1.7 Hz, 1H), 10.33 (s, 1H). ^13^C NMR (100 MHz, DMSO-d_6_) *δ* 33.01, 100.84, 109.91, 112.89, 116.37, 123.92, 128.21, 130.74, 131.27, 131.41, 134.16, 135.77, 149.07, 152.27, 164.03.

### 2.2. Monoamine oxidase enzyme assay

The inhibitory activity of MAO-A, B enzyme of the compounds was evaluated based on previously described method^25^. The MAO activity was measured in relative fluorescence units (RFUs) evoked by peroxidase-catalyzed oxidation of Amplex Red^®^ to resorufin, in which H_2_O_2_ generated by MAO reaction was used as the electron donor. Human recombinant MAO-A (hMAO-A) and MAO-B (hMAO-B) enzyme expressed in insect cells were obtained from Sigma-Aldrich (St. Louis, MO). The 2 μL of test compound in DMSO (final concentration: 1 nM to 10 μM) was treated with 98 μL of hMAO enzyme solution in 50 mM sodium phosphate buffer (pH 7.4, final protein amounts: ∼1.25 μg protein/well for MAO-A and ∼2.5 μg protein/well for MAO-B) on 96-well black plate and incubated for 15 min at 37 °C. Then, 100 μL of reaction working solution which is mixed solution of 400 μM Amplex Red^®^ (Cayman, Ann Arbor, MI, final concentration: 200 μM), 2 U/mL horseradish peroxidase (Sigma-Aldrich, St. Louis, MO, final concentration: 1 U/mL) and 2 mM substrate (*p*-tyramine for MAO-A, benzylamine for MAO-B, Sigma-Aldrich, St. Louis, MO, final concentration: 1 mM) in 50 mM sodium phosphate buffer (pH 7.4) were added and incubated for 20 min at 37 °C in the dark. The fluorescent intensity was quantified using a microplate reader (SpectraMax^®^*i*3, Molecular Devices, Sunnyvale, CA) with an excitation at 545 nm and an emission at 590 nm. The 50% inhibitory concentrations (IC_50_) of compounds were determined as the mean ± SEM in triplicate from the dose–response inhibition curves using SigmaPlot^®^ 13.0 (Palo Alto, CA).

### 2.3. Kinetic study of MAO-B inhibition mode

The type of MAO-B inhibition of compound **4e** was determined by the Michaelis–Menten kinetic study. The catalytic rates of hMAO-B enzyme in the absence or in the presence of three different concentrations (10, 30, and 100 nM) of compound **4e** were measured at seven different concentrations of the benzylamine (0.065, 0.125, 0.25, 0.5, 1, 2, and 4 mM). The corresponding progression curves and the Lineweaver–Burk plots were generated using GraphPad Prism 7 (La Jolla, CA). The maximal velocity (*V*_max_), the Michaelis constant (*K*_m_), and the inhibition constant (*K*_i_) were calculated using SigmaPlot^®^ 13.0 (Palo Alto, CA).

### 2.4. Molecular modelling study

Crystal structure of MAO-A (PDB ID: 2Z5X)^48^ and MAO-B (PDB code: 2V5Z)^31^ protein downloaded from protein data bank (www.pdb.org). MAO-B structure (PDB code: 2V5Z) is complexed with safinamide and MAO-A structure (PDB ID: 2Z5X) complexed with harmine (7-methoxy-1-methyl-9h-beta-carboline). Protein structures prepared using the protein preparation wizard^49^ of Schrodinger 2018 suite of the package (Schrödinger LLC, New York, NY) at the default setting and 7.4 pH value. All the inhibitors were sketched using ChemDraw Professional 16.0 and saved as structure data file format and imported to Ligprep module. Ligprep module of Schrodinger was used to prepare all ligands, and further geometry optimized. Re-docking X-ray ligands confirmed the reproducibility of the docking program (data not shown). All minimised conformations of ligands were docked into the safinamide binding site using Glide's standard precision module^50^ and generated 10 poses for each ligand. The docking figures were rendered using the Discovery Studio Client 2019 package (Dassault Systèmes; BIOVIA. Discovery Studio Modeling Environment; Release 2019; Dassault Systèmes: San Diego, CA, 2019). We selected the docked poses with more negative Glide docking scores and significant interactions with MAO-B protein.

## 3. Results and discussion

### 3.1. Chemical synthesis

**Scheme 1.**
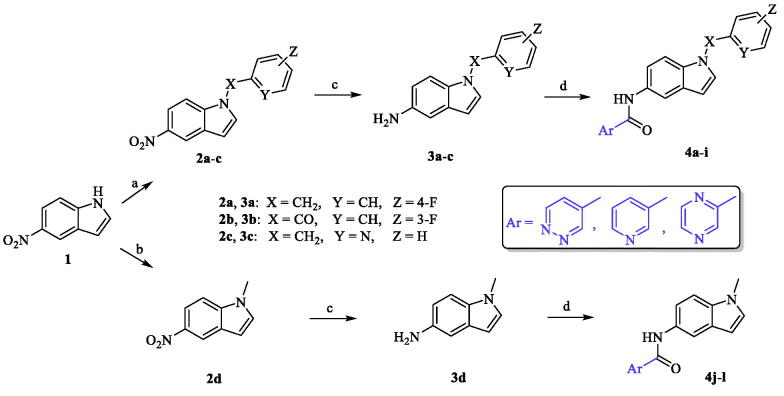
Reagents and conditions: (a) appropriate halide derivative, NaH (60% in oil), DMF, 0–100 °C, 24 h; (b) dimethyl carbonate, K_2_CO_3_, DMF, reflux, 3 h; (c) NH_4_Cl, Fe, EtOH/H_2_O, reflux, 1 h; (d) appropriate heteroaryl carboxylic acid, DIPEA, HATU, DMF, MW, 116 °C, 45 min.

The newly synthesized target compounds **4a**−**l** were prepared as outlined in Scheme 1. Starting from the commercially available 5-nitroindole (**1**), substitution on position N1 of the indole core was carried out using 4-fluorobenzyl bromide for **2a**, 3-fluorobenzoyl chloride for **2b**, and 2-(chloromethyl)pyridine hydrochloride for **2c**, in presence of NaH (60% in oil) and dimethylformamide (DMF) solvent to yield analogues **2a**−**c**^39–41^. Dimethyl carbonate reagent was used as a methyl group donor to the amine moiety in compound **1** by heating both in presence of potassium carbonate as a basic catalyst to give compound **2d**^42^. Preparation of intermediates **3a**−**d**, required for synthesis of the final analogues, was performed by reduction of 5-nitro group in **2a**−**d** using iron powder as a dissolving metal in a slightly acidic medium (ammonium chloride)^51^. With intermediates in hand, the last step in the synthetic route was achieved *via* amide coupling reactions between the free amine groups in **3a**−**d** and the appropriate heteroaryl carboxylic acid in presence of DIPEA and HATU to afford the target small molecules **4a**−**l** (full chemical structures, Table 1).

**Table 1.** Inhibitory effects of the synthesized compounds against MAO-B.

^a^ Inhibition percent of MAO-B at single dose of 10 µM of the prepared compound.

### 3.2. MAO-B assay

#### 3.2.1. Inhibition of MAO-B by the prepared compounds 4a–4l at single dose of 10 µM

The target compounds **4a**−**l** were biologically evaluated for their potential inhibitory activities over human MAO-B recombinant enzyme. The enzyme inhibition assay of the tested compounds was conducted using Amplex^®^ Red reagent technology which offers quantitative enzyme activity sensitive detection with extended dynamic range compared to classic colorimetric oxidase assays in addition to its advantage of the fluorescence emission outside the range of compound autofluorescence (if any). Since Amplex^®^ Red reagent is a colourless substrate able to react with H_2_O_2_ with a 1:1 stoichiometry to yield highly fluorescent resorufin, the elicited inhibitory activities of the tested compounds over MAO-B were evaluated *via* spectrophotometrical measurement and the fluorescence rate of the resorufin dye formation was detected. As mentioned previously, all the target compounds were designed to incorporate the same amide moiety in **VI** and **VII** at position 5 of the indole scaffold connecting one of three different heteroaromatic cycles with the indole core which is substituted with either aromatic or aliphatic moiety. First, a set of nine compounds (**4a**−**i**, series A) was prepared possessing varied aromatic functional groups on N1 of the indole. By analyzing the obtained preliminary biological results of series A (Table 1), it was confirmed that our rational design of retaining the original amide spacer of **VI** and **VII** with the N-substituted indole core is still able to yield even more active MAO-B inhibitors (**4b** and **4e**). In addition, we noticed that the benzyl and benzoyl moieties, bearing the electron-withdrawing 3- or 4-fluoro groups in **4a**−**f**, exerted better inhibition than the pyridylmethyl substituent (**4g**−**i**). Also, while modest inhibition values were elicited by analogues **4a**, **4c**, **4d**, and **4f** possessing either benzyl or benzoyl moiety on the indole ring (13.15 ± 3.32%, 11.24 ± 0.24%, 19.08 ± 0.47%, and 30.29 ± 0.24%, respectively), it was noted that only two heteroaryl cycles (pyridine and pyridazine) attached to the amide spacer were represented in the chemical structures of these four compounds. Interestingly, analogues **4b** and **4e** having the 2-pyrazinyl moiety and 4-fluorobenzyl moiety or 3-fluorobenzoyl moiety, respectively, were the most active compounds in this series with inhibition values of 88.49 ± 0.13% for **4b** and 66.39 ± 0.79% for **4e**. However, as mentioned, when the substitution on N1 of the indole scaffold was changed to the pyridylmethyl group, the inhibitory activity over MAO-B was dramatically decreased to disclose low active derivatives **4g**, **4h**, and **4i** (1.83 ± 0.75%, 2.38 ± 0.66%, and 1.81 ± 0.85%, respectively). It is also noteworthy to mention that the three different heteroaryl cycles incorporated in our design (pyridazine, pyrazine, and pyridine) were represented in each of the last three compounds. Thus, taking into consideration the significant difference of the biological activity between compounds **4b** and **4h** possessing the same heteroaryl cycle (2-pyrazinyl), we have concluded that not only the type of the heteroaryl cycle attached to the amide linker is an essential factor to design a highly active MAO-B inhibitor, but also the nature of N1 substituent of the indole core; both are necessary elements to acquire a higher MAO-B inhibitory effect.

Next, we totally changed the aromatic substitution on N1 of the indole moiety to methyl group to acquire series B (**4j**−**l**). It was noted that the inhibitory activities elicited by compounds **4j**−**l** significantly increased compared to those of **4g**−**i** having pyridylmethyl group (9.52 ± 0.61%, 49.68 ± 0.68%, and 11.57%±0.9 for compounds **4j**, **4k**, and **4l**, respectively). It was important to keep the three heteroarylcycles fully represented in this series (**4j**−**l**) in order to outline a fine-tuned interpretation about their inhibitory effect over MAO-B. Interestingly, compound **4k** possessing 2-pyrazinyl ring attached to the amide linker showed higher inhibitory effect than compounds **4j** and **4l**, which reconfirms the value of the 2-pyrazinyl heterocycle as essential factor to obtain higher inhibitory effect over MAO-B.

#### 3.2.2. Dose dependent assay of the most active analogues 4b and 4e over MAO-B

**Figure 3.**
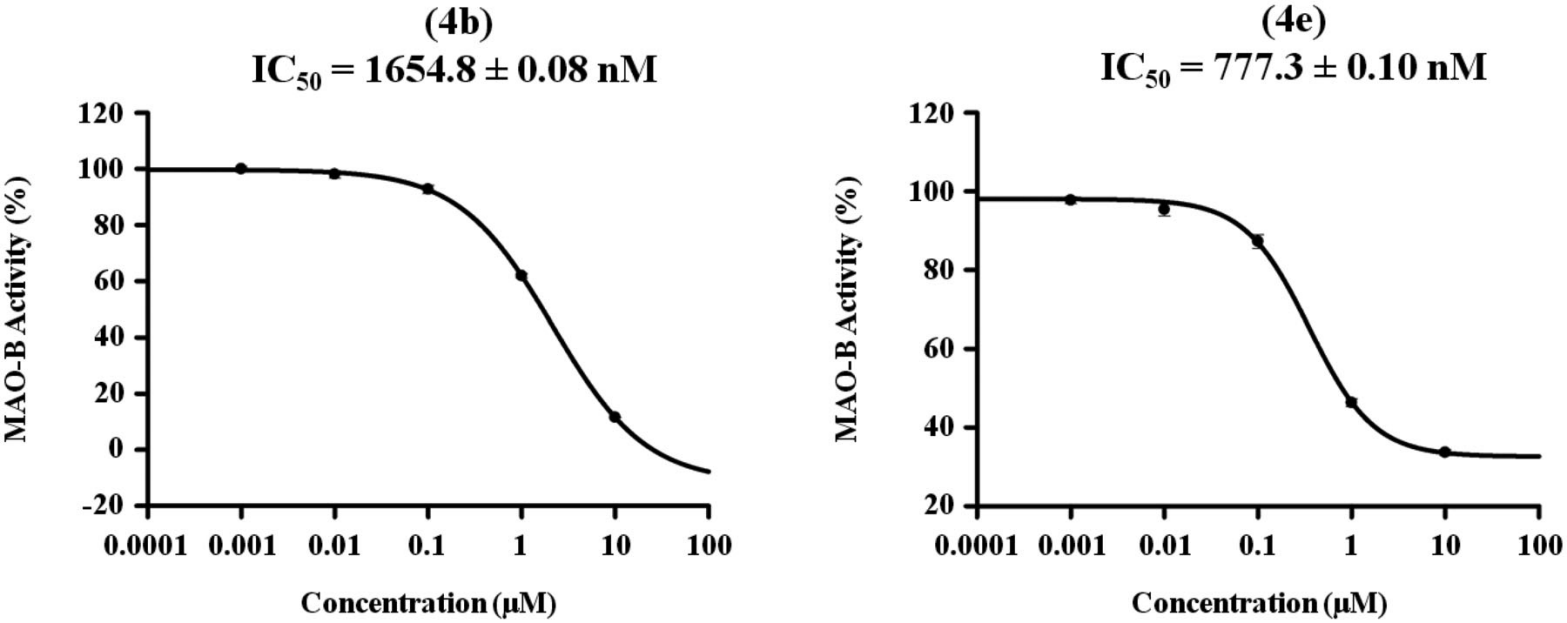
Dose dependent assay of compounds **4b** and **4e** over MAO-B.

The inhibitory potencies (IC_50_ values) of compounds producing inhibition of MAO-B activity by more than 50% were determined using five doses assay with 10, 1, 0.1, 0.01, and 0.001 μM concentrations of the tested compounds. Consequently, analogues **4b** and **4e** were further evaluated to determine their IC_50_ over MAO-B in triplicate from the dose response inhibition curves using Sigma-Plot software version 13.0 as indicated in Figure 3. While compound **4b** exerted low micromolar IC_50_ value of 1654.8 ± 0.08 nM, a higher potency in sub-micromolar range elicited by analogue **4e** (777.3 ± 0.10 nM).

#### 3.2.3. Selectivity assay of compounds 4b and 4e

It is known that non-selective (MAO-A/B combined) inhibitors lead to several problems when taken concomitantly with tyramine-containing foods (such as cheese) since the drug's inhibition of MAO-A causes a dangerous elevation of serum tyramine levels, which can cause hypertensive symptoms^52^. On the other side, selective MAO-B inhibitors can bypass this problem by preferentially inhibiting MAO-B. Thus, to check the selectivity of the most active derivatives for the MAO-B isoform, a selectivity assay was performed by testing analogues **4b** and **4e** over MAO-A at a high dose concentration of 100 µM followed by detection of their IC_50_ values. As presented in Table 2, both derivatives exhibited very weak inhibitory effect over MAO-A even with a highly concentrated dose of 100 µM (12.95 ± 1.09% and 4.76 ± 0.59% for **4b** and **4e**, respectively). To calculate the selectivity index (SI: the selectivity for the MAO-B isoform given as the ratio of IC_50_ (MAO-A)/IC_50_ (MAO-B)), IC_50_ values of both compounds have been identified and found to be more than 100 µM. Consequently, the selectivity indices were calculated as illustrated in Table 2. While compound **4b** showed selectivity index > 60 for MAO-B isoform, the most active derivative **4e** has a higher SI value (>120). Thus, compared to the modest selectivity index of rasagiline (**II**, calculated SI > 50^33^) both **4b** and **4e** were more selective for MAO-B. Accordingly, compound **4e** was selected for further evaluation to define its kinetic mode of interaction with the human MAO-B enzyme.

**Table 2.** Inhibitory effects of the compounds **4b** and **4e** against MAO-A.

Compound	MAO-A % inhibition^a^	MAO-A (IC_50_, µM)	SI^b^
**4b**	12.95 ± 1.09	>100	>60
**4e**	4.76 ± 0.59	>100	>120

^a^ Inhibition percent of MAO-A at single dose of 100 µM of the prepared compound.

^b^ SI = selectivity index, the selectivity for the MAO-B isoform and is given as the ratio of IC_50_ (MAO-A)/IC_50_ (MAO-B).

#### 3.2.4. Kinetic study to define the interaction mode of compound 4e with MAO-B

**Figure 4.**
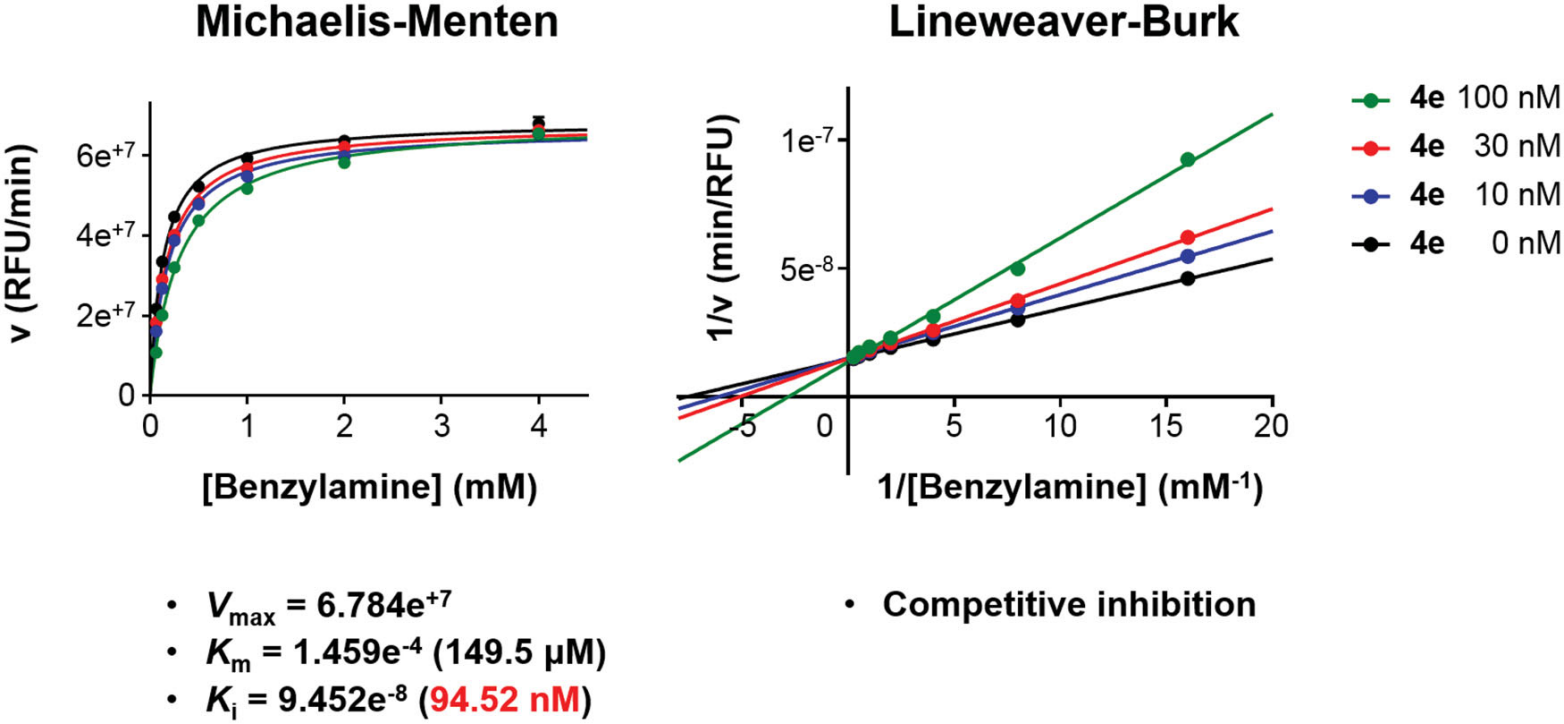
Type of inhibition of MAO-B by compound **4e**. The catalytic rates were measured at different concentrations of benzylamine (0.065, 0.125, 0.25, 0.5, 1, 2, and 4 mM) in the absence and in the presence of different concentrations (10, 30, and 100 nM) of compound **4e**. The *V*_max_, *K*_m_, and *K*_i_ values were calculated using SigmaPlot^®^.

Detailed substrate-dependent kinetic experiments were carried out to examine the mode of MAO-B inhibition of compound **4e**. The initial rates of MAO-B inhibition in the absence and presence of **4e** were measured at various concentrations of benzylamine, a selective substrate for MAO-B, and both Michaelis–Menten and the Lineweaver–Burk plots are depicted in Figure 4. The Lineweaver–Burk plots for three different concentration of **4e** were linear and intersected at the *y*-axis. The maximal velocity (*V*_max_), the Michaelis constant (*K*_m_), and the inhibition constant (*K*_i_) were calculated using SigmaPlot^®^ (Palo Alto, CA) (*V*_max_ = 6.784e^+7^, *K*_m_ = 1.459e^−4^, and *K*_i_ = 9.452e^−8^). Hence, it can be stated that compound **4e** is competitive, and accordingly, reversible MAO-B inhibitor.

### 3.3. Molecular docking

**Figure 5.**
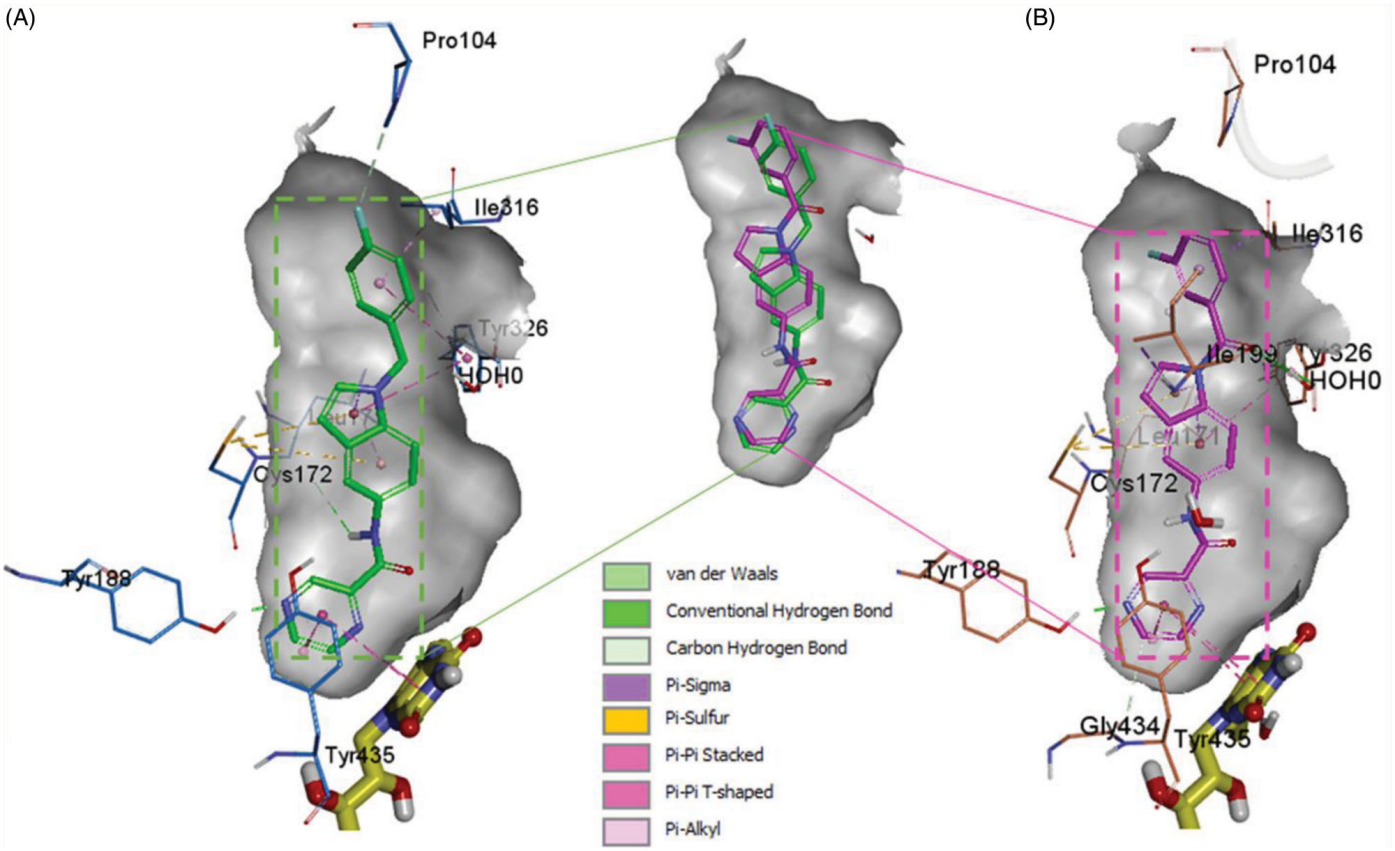
The docked model of the most active compounds **4b** (A) and **4e** (B) into MAO-B binding pocket.

**Figure 6.**
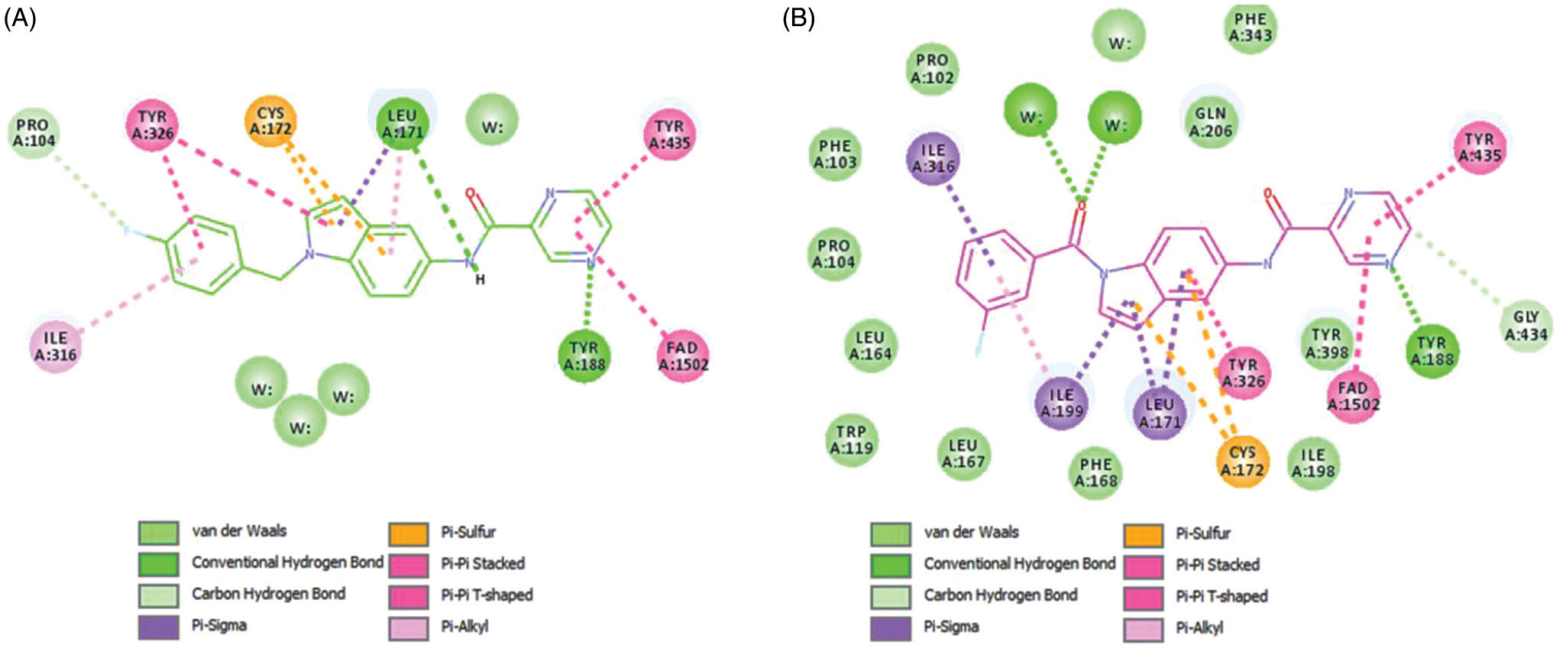
Two-dimensional (2D) interaction model for **4b** (A) and **4e** (B) with MAO-B.

In order to provide deeper insights into the SAR of the new series, the most active compounds **4b** and **4e** were selected to be docked into the active binding region of MAO-B. Therefore, the docking studies were conducted in order to determine the *in silico* binding modes of these compounds, evaluate the contribution of the substitution on N1 of the indole moiety, understand the essential incorporation effect of 2-pyrazinyl heterocycle to MAO-B enzyme activity, gain insights for the selectivity of this class of compounds for MAO-B isoform, and finally to prove the non-covalent reversible binding of this class of inhibitors to MAO-B. The X-ray crystal structures of MAO-B (PDB ID: 2V5Z)^31^ were obtained from Protein Data Bank (www.pdb.org). The docked model of the most active compounds **4b** and **4e** is demonstrated in Figure 5; **4b** (A) and **4e** (B), while Figure 6 shows their two-dimensional (2D) interaction model; **4b** (A) and **4e** (B).

For compound **4b** (Glide score −10.62) (Figure 5(A)), the central indole ring interacted *via* π–π-stacking, π–sulphur, and π–sigma interactions with Tyr326, Cys172, and Leu171, respectively. The pyrazine ring interacts *via* an H-bond, π–π stacking, and T-shaped π–π-stacking interactions with Tyr188, and Tyr435, and fused ring of FAD, respectively. The amide “NH” forms an H-bond with the main chain carbonyl of Leu171 while 4-fluorobenzyl moiety forms π–sigma and π–π-stacking interactions with the Ile316 and Tyr326, respectively. It was noticed that Fluorine atom at the para position contributed to week carbon–hydrogen bond with Pro104. Interestingly, a similar binding pose of **4e** (Glide score −10.79) was observed in the binding pocket of MAO-B with a shifted orientation (Figure 5(B)). The indole ring packed at the centre of the binding cavity having π-sigma interactions with Ile199, Leu171, and T-shaped π stacking interaction with Tyr326, respectively. Cys172 holds the indole ring by two π–sulphur bonds. 3-fluorobenzoyl group is shifted towards a more hydrophobic pocket in line with Pro104, Ile199, Ile316, and Tyr326. The oxygen of this carbonyl spacer involved in two H-bond interactions with a water molecule (distance ∼2.6 Å). Whereas, the CH_2_ group in **4b** does not involve in any H-bond interactions. This could highlight the higher potency of **4e** over **4b**. The carbonyl linker at N1 position has improved the binding efficacy as well as potency. Pyrazine ring placed near to FAD ring and showed two π–π stacking bonds with FAD and Tyr435. The nitrogen atom in pyrazine ring (at 4-position) established a strong H-bond bond with Tyr188 (distance 1.9 Å).

From the docked poses of **4b** and **4e**, it was concluded that to get higher potency, compounds should possess 1,4-pyrazine and *m*-fluorobenzoyl moieties. From the SAR study, we can see that compounds **4a** and **4c** possess pyridazine and pyridine rings show 13.15% and 11.24% of MAO-B inhibition compared with **4b** (88.49%) and **4e** (66.39%). In case of compounds **4d** and **4f**, both have *m*-fluorobenzoyl substitution at indole “N,” but **4f** shows more significant inhibition of MAO-B. It also suggests that the pyridine ring is more tolerable than pyridazine. On the other side, when the fluorobenzyl is replaced by 2-methylpyridine (**4g**, **4h**, and **4i**), a significant decrease in the % MAO-B inhibition was noticed. The reason for the reduced MAO-B inhibitory activity could be the relative higher polarity of 2-methylpyridine compared to the fluorobenzyl moiety. The surrounding region at this substitution is highly hydrophobic lined by the residues Pro104, Trp119, Leu164, Leu167, Ile199, and Ile316. The incorporation of more hydrophobic ligand substitution may enhance the % MAO-B inhibition, and polar substitution might lead to a further reduction in % MAO-B inhibition. Further, removal of *p*-fluorophenyl from the compounds **4a**, **4b**, and **4c** obtained compounds **4j**, **4k**, and **4l** displayed a reduction in % MAO-B inhibition. This suggests the importance of *p*-fluorophenyl moiety in the MAO-B inhibition, because this part of ligand is docked at the upper pocket of MAO-B, where it holds π–π-stacking, π–sulphur, π–sigma interactions with Tyr326, Cys172, and Leu171. Removal of such interactions (**4j**–**4l**) results in deterioration of the % inhibition of MAO-B.

**Figure 7.**
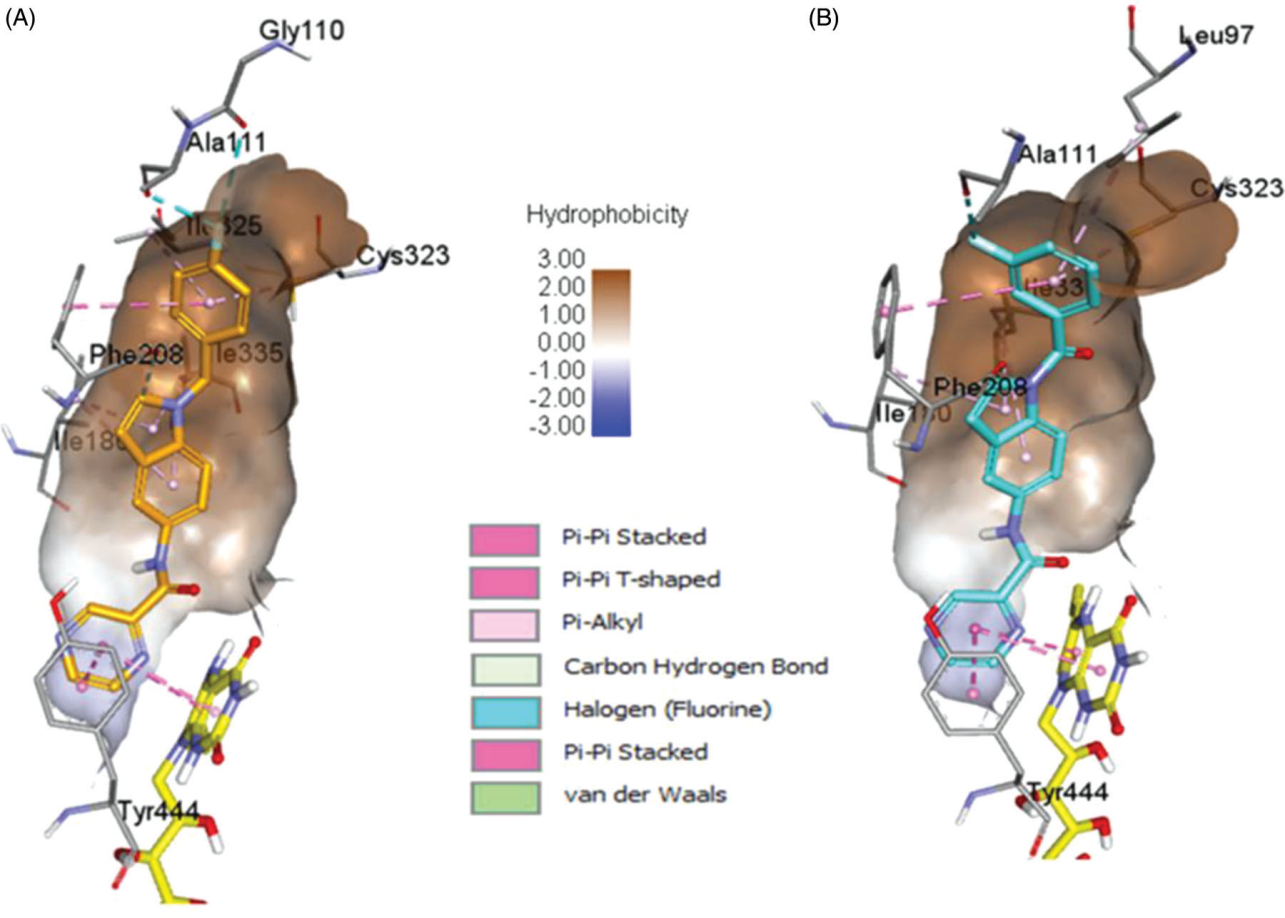
Binding orientation of ligand **4b** and **4e** inside the MAO-A protein structure. Ligand **4b** (A) and **4e** (B) shown in orange and blue colour stick format. FAD molecule shown in yellow stick format. Hydrophobic surface area shown around the ligand.

**Figure 8.**
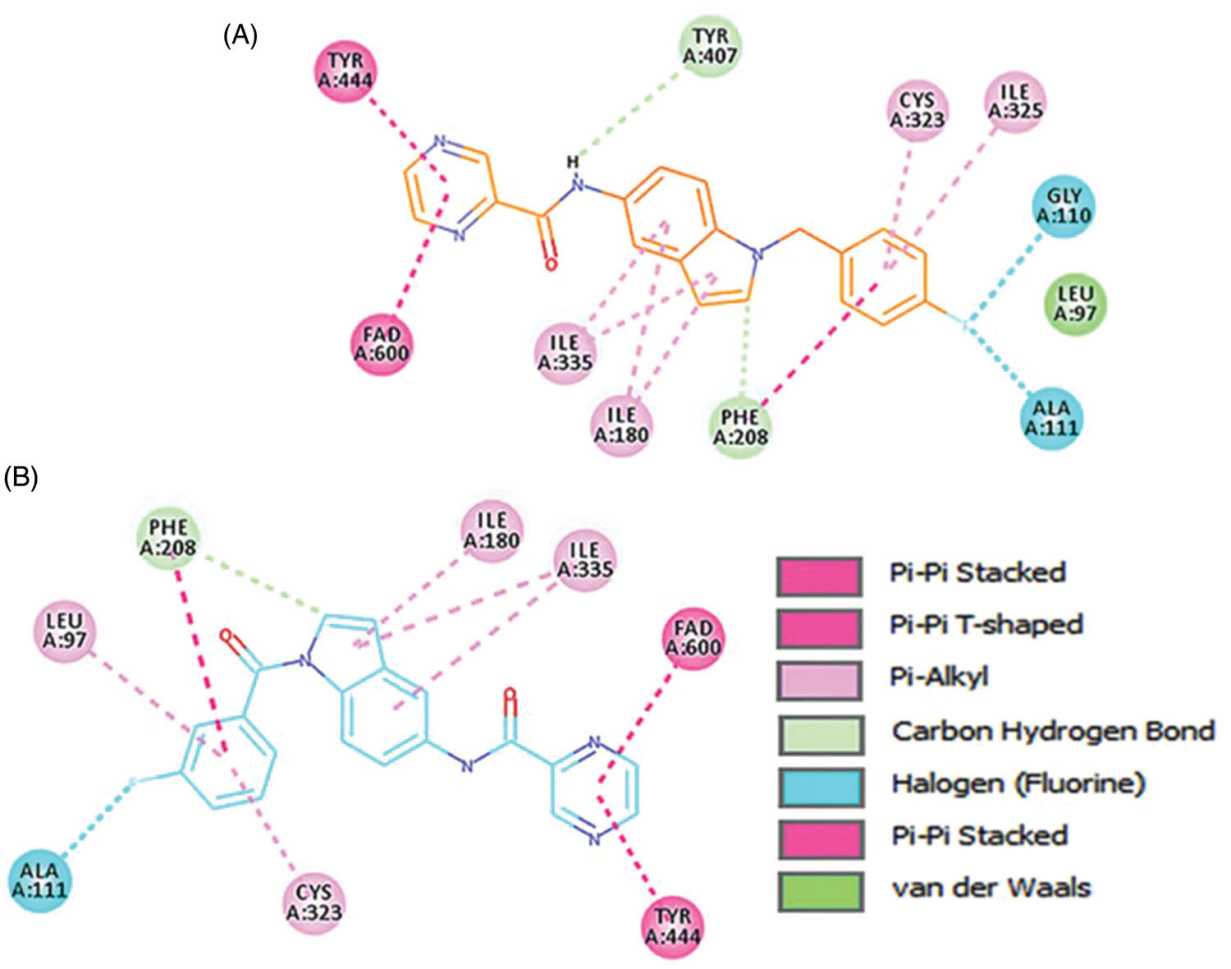
2D interactions of ligands **4b** and **4e** inside MAO-A binding pocket. **4b** (A) and **4e** (B) ligands shown in orange and blue colour, respectively.

**4b** and **4e** ligands were docked inside the MAO-A protein (PDB ID: 2Z5X)^48^. For **4b** ligand (glide score −6.65), the pyrazine ring formed strong π–π-stacked interactions with FAD and Tyr444 (Figures 7 and 8). The amide linker showed no H-bond interactions with the surrounding residue; it established only one week carbon hydrogen bond with Tyr407. The central indole ring has week π–alkyl interactions with Ile180, Ile335. Fluorobenzyl group has strong π–π-stacked and week π–alkyl contact with Phe208 and Cys323, Ile325, respectively. Fluorine substitution on para position has two halogen interactions with Gly110, and Ala111. Similarly, ligand **4e** (glide score −4.20) has a week π–alkyl interaction between indole core and Ile180, and Ile335 (Figures 7 and 8). The amide linker had no strong interactions with the surrounding residues. The pyrazine ring showed π–π-stacked interactions with Tyr444 and π–π-T-shaped contact with FAD. Upper hydrophobic pocket fluorobenzyl group showed π–alkyl interaction with Leu97, Cys32, and π–π-stacked interactions with Phe208. Fluorine atom has only one-halogen bond interactions with Ala111. Carbonyl linker did not show any contribution to protein-ligand interactions. Compared to MAO-B protein, ligands **4e** and **4b** have weaker interaction patterns inside MAO-A binding pocket.

Overall, MAO-A and MAO-B proteins have a higher sequence identity and similarity. The two crystal structures of MAO-A (PDB ID: 2Z5X) and MAO-B (PDB ID: 2V5Z) have 73.3% sequence identity. It was noted that the binding site residues are also very similar except for some residues. Similar and variable binding site residues of MAO-B/MAO-A are listed as follows: Leu88/Leu97, Pro102/Ala111, Gly101/Gly110, Pro104/Pro113, Leu171/Ile180, Cys172/Asn181, Tyr188/Tyr197, Ile199/Phe208, Thr314/Cys323, Ile316/Ile235, Tyr326/Ile335, Tyr398/Tyr407, and Tyr435/Tyr444. Important Leu171 (MAO-B) residue involves in H-bond interactions with amide linker of the ligand and reliable π–sigma contacts with the central indole ring, which is replaced with Ile180 (MAO-A) that only constitutes few week pi-alkyl interactions with the ligand. Another side similar aromatic residues Tyr188/Tyr197; Tyr188 (MAO-B) has strong H-bond with pyrazine nitrogen while Tyr197 in MAO-A does not show any promising interaction with the ligand moieties. Cys172 in MAO-B has also significant π-sulphur interaction with the central indole ring, while in MAO-A protein, it was replaced by Asn181 which does not interact at all with the ligand molecules. Overall, in the docking study, **4b** and **4e** ligand showed higher docking score inside MAO-B protein. Additionally, the abundant aromatic interactions (π–π, π–sigma, and π–sulphur) and strong H-bond interactions mediated by pyrazine nitrogen, carbonyl oxygen, and carboxamide nitrogen inside MAO-B protein make ligands **4b** and **4e** to be more selective towards MAO-B protein.

## 4. Conclusions

In summary, we present simple and convenient chemical synthetic route using microwave conditions towards synthesis of new promising MAO-B inhibitors. Our results investigated *in vitro* MAO-B inhibitory activity of two indole-based series originally designed from two indazole-containing lead compounds (**VI** and **VII**); by retaining their original amide spacer while applying scaffold modification into an indole core. Moreover, various substitution patterns were incorporated on the free NH group of the indole aiming to obtain a new lead with potential MAO-B inhibitory activity. Interestingly, amongst all members, compound **4e** displayed a promising profile of high potency, selectivity, in addition to a competitive MAO-B mode of action. As a result, our group is currently performing further optimization studies of compound **4e** focussing on computer-aided lead optimization to advance more potent indole-based derivative as a step towards a new potential clinical candidate for MAO-B inhibition.
